# Case Report: Adjunctive hyperbaric oxygen therapy in pediatric glans reimplantation following penile trauma

**DOI:** 10.3389/fsurg.2025.1626866

**Published:** 2025-07-24

**Authors:** Ramsey Amoudi, Gabriel Carreno-Galeano, Katie Canalichio

**Affiliations:** ^1^ School of Medicine, University of Louisville, Louisville, KY, United States; ^2^ Department of Urology, University of Louisville School of Medicine, Louisville, KY, United States; ^3^ Norton Children’s Urology, Norton Children’s Hospital, Louisville, KY, United States

**Keywords:** case report, pediatric penile trauma, hyperbaric oxygen therapy, partial glans avulsion, tissue reimplantation, graft viability

## Abstract

Pediatric penile trauma is a rare urological emergency, and evidence-based guidelines for complex tissue salvage are limited. We report the case of an eight-year-old boy who presented six hours after sustaining a partial ventral glans avulsion. The patient underwent evaluation under general anesthesia followed by delayed surgical reimplantation of the avulsed glans tissue. As an adjunct to surgery, he received ten consecutive sessions of hyperbaric oxygen therapy (HBOT). Post-treatment follow-up demonstrated complete graft integration, excellent cosmetic outcome, preserved urinary function, and no complications. This case underscores the potential of HBOT to enhance graft viability and recovery in the setting of delayed reimplantation. Drawing on prior experience with graft-based reconstruction in complex hypospadias reoperations, HBOT may serve as a valuable adjunct in pediatric penile trauma involving complex tissue loss, offering a promising strategy to improve outcomes in similarly challenging reconstructive scenarios.

## 1 Introduction

Penile trauma in the pediatric population is rare but can result in significant tissue loss requiring complex reconstruction (1). In severe cases, avulsion injuries may necessitate delayed grafting due to loss of viable skin or tissue (2). We report a case of partial glans avulsion in an 8-year-old boy, in which the avulsed portion of the glans was successfully reimplanted approximately six hours after the injury. The unique aspect of this case lies in the use of adjunctive hyperbaric oxygen therapy (10 sessions) to enhance graft viability. This approach resulted in complete reintegration of the tissue without any necrosis or loss, despite the delayed reimplantation.

## 2 Case description

We present the case of an 8-year-old boy who sustained a penile injury under unclear circumstances. At the time of the incident, the child was unsupervised. His grandparents were present in the home but were not directly attending to him, and his parent was away. According to the history provided by the family, the injury occurred when the toilet seat accidentally fell while the child's penis was resting on the toilet seat ring. This reportedly resulted in a partial avulsion of the ventral portion of the glans penis.

## 3 Initial presentation and clinical timeline

**Figure 1:**
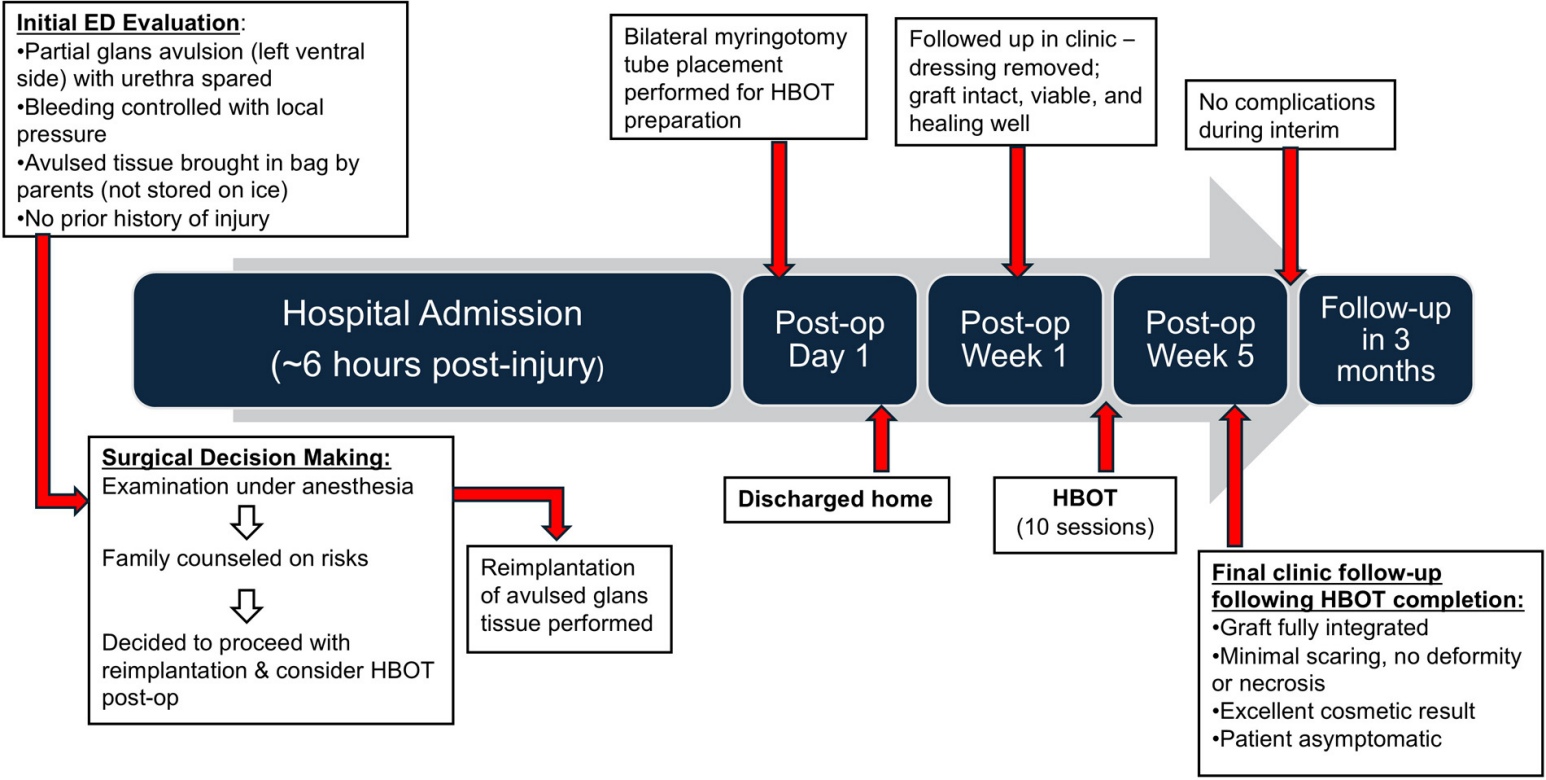
Timeline of key clinical events from initial injury to follow-up. ED, emergency department; HBOT, hyperbaric oxygen therapy.

**Figure 2:**
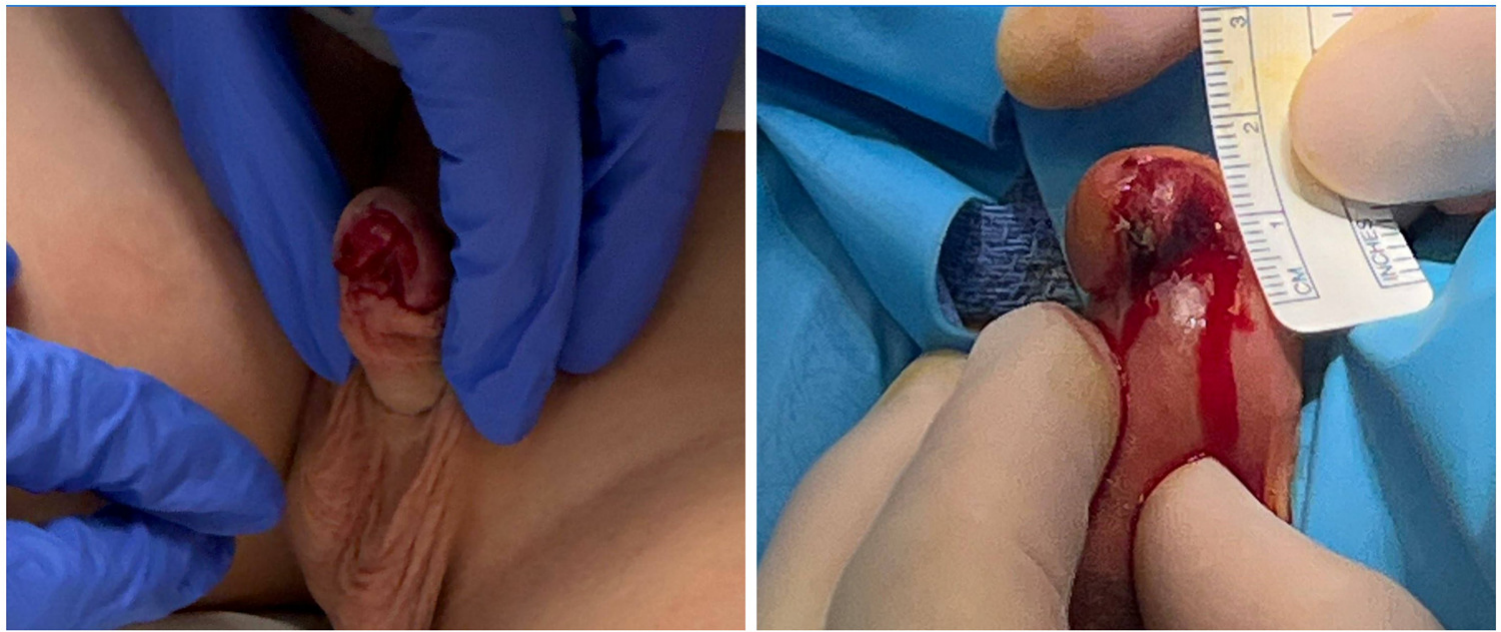
**(A**, *left panel***)** preoperative image showing partial avulsion of the ventral, left-sided glans. Urethral involvement could not be fully assessed on external examination in the emergency department. **(B**, *right panel***)** Intraoperative image showing the ventral glans avulsion with a measuring tape in view for size reference.

On initial evaluation in the emergency department approximately six hours after the injury, the patient was found to have a partial avulsion involving nearly the entire left ventral glans. A timeline of the clinical events is summarized in Figure 1. At the time of assessment, the urethra and urethral meatus could not be clearly evaluated. Active but slow bleeding was present and was successfully controlled with local pressure (Figure 2A).

The patient's father brought the avulsed glans tissue to the emergency department in a Zip-loc bag; notably, it had not been stored on ice. The patient had no significant past medical history.

## 4 Diagnostic assessment, details on the therapeutic intervention, follow-up and outcomes

Following multidisciplinary evaluation and detailed counseling with the family, the decision was made to proceed to the operating room for examination under anesthesia, cystoscopy, and definitive management. Intraoperatively, physical examination confirmed an intact urethral meatus and exposed spongy tissue of the corpus spongiosum at the site of avulsion. The medial extent of the injury reached the left lip of the urethral meatus (Figure 2B). Cystoscopy confirmed an intact urethral lumen without evidence of internal injury.

Surgical options included primary wound closure or attempted reimplantation of the avulsed glans tissue, despite the delayed presentation. The family was extensively counseled on the uncertain viability of the graft and the associated risks, including graft necrosis, failure, scarring, cosmetic deformity, glans shrinkage, and potential long-term effects on penile development and function, particularly with future erections.

Based on experience from reoperative hypospadias repair, the potential benefit of adjunctive postoperative hyperbaric oxygen therapy (HBOT) was also discussed. After careful consideration of the benefits, risks, and logistics involved, the family consented to proceed with HBOT in the event that glans reimplantation was performed.

**Figure 3:**
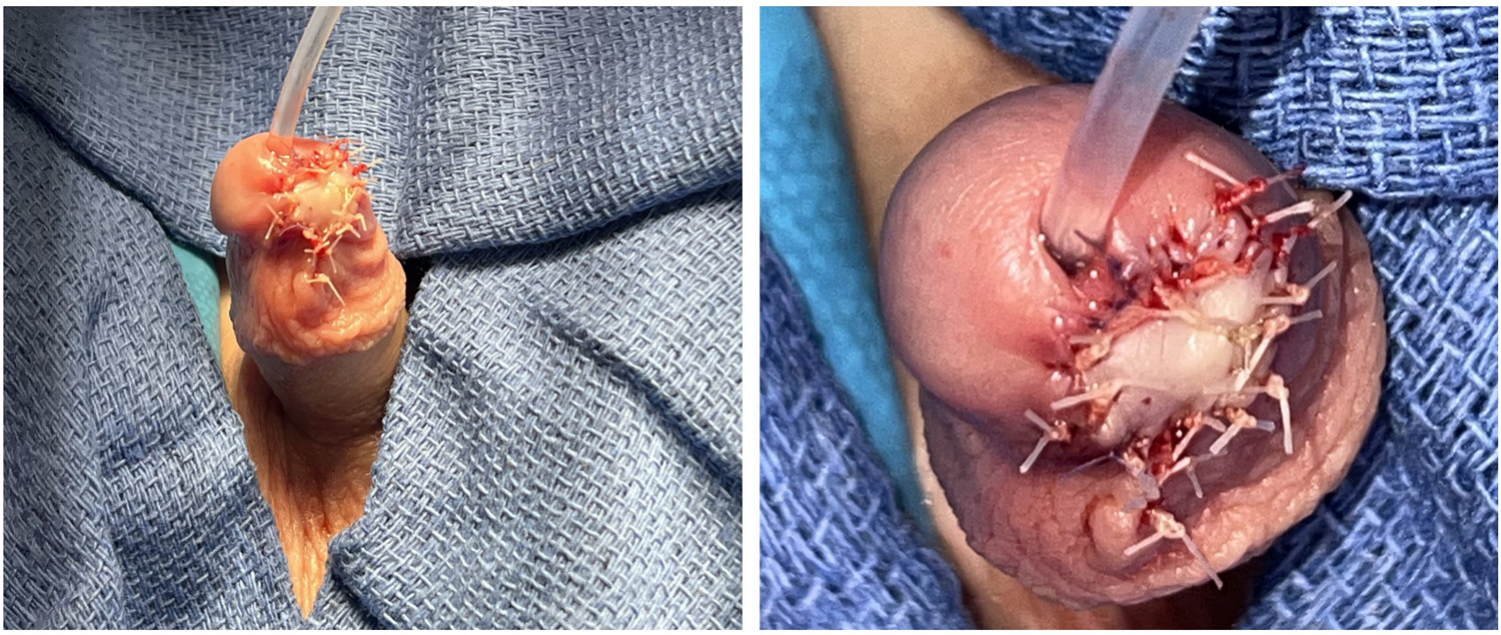
**(A**, *left panel***)** immediate postoperative appearance following glans reimplantation (front-lateral view). **(B**, *right panel***)** Immediate postoperative appearance (top-down view) showing catheter placement and an intact urethral meatus without involvement in the injury.

Intraoperatively, both the wound bed and the avulsed glans tissue were thoroughly irrigated with normal saline and Irricept. Reimplantation was then performed using interrupted 5–0 Vicryl sutures to secure the glans tissue, with 7–0 Vicryl sutures used specifically to reapproximate the avulsed tissue to the left lip of the urethral meatus to minimize scarring. A single mattress suture was used to secure the graft to the glans wound bed. An 8 French Foley catheter was placed, and a standard hypospadias dressing was applied (Bacitracin, Telfa, Mastisol, and Tegaderm) (Figure 3). *Note: the hypospadias dressing is not shown in the figure*.

The following day, the patient underwent bilateral myringotomy tube placement in preparation for HBOT and was subsequently discharged home. HBOT was initiated approximately one week after discharge, following clearance by otolaryngology.

**Figure 4:**
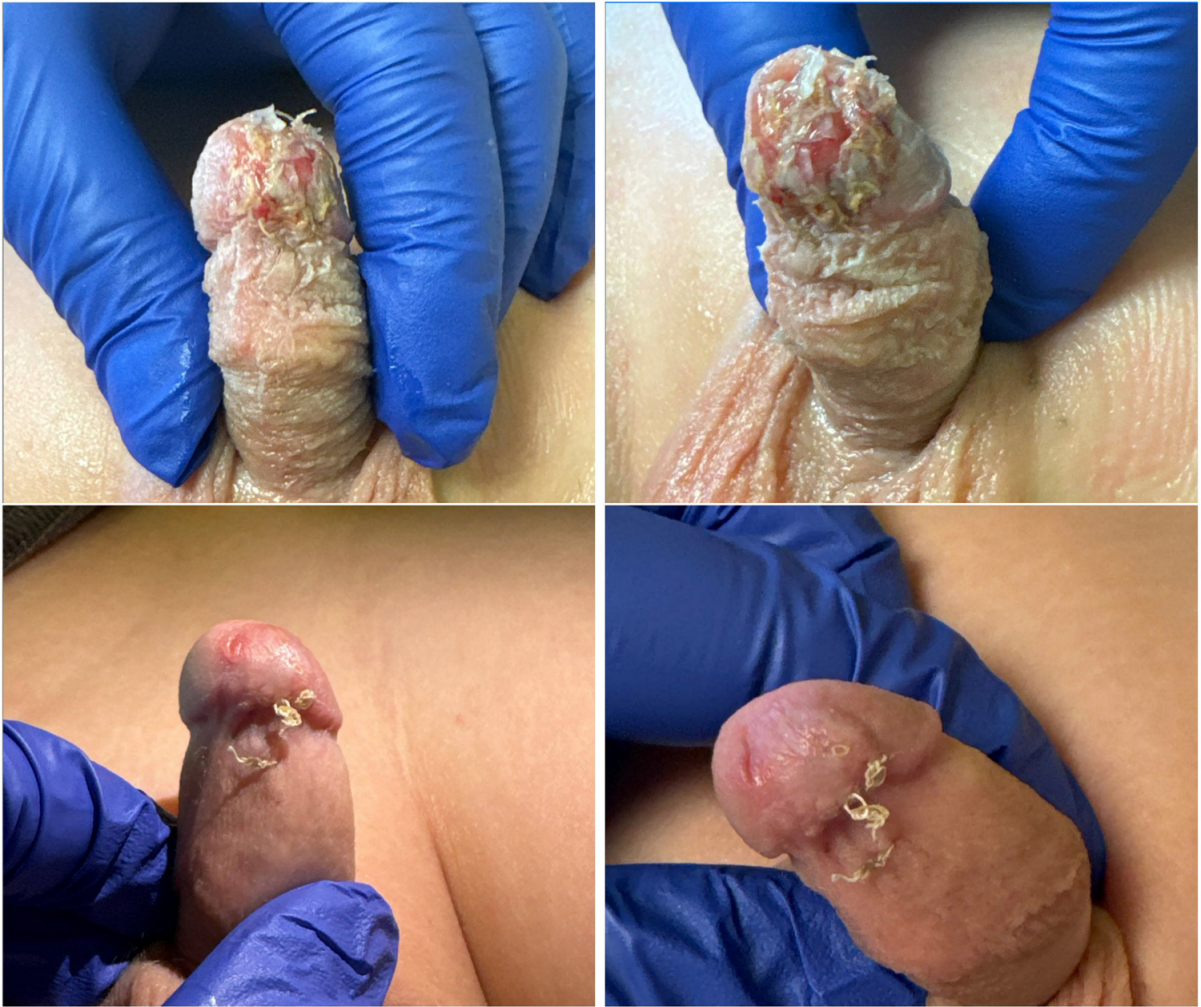
**(A**, *top left*; **B**, *top right***)** One week after surgery, immediately following removal of the hypospadias-style dressing (not shown). The graft appears viable with early signs of healing. **(C**, *bottom left*; **D**, *bottom right***)** Five weeks postoperatively, showing complete tissue integration, excellent cosmetic result, minimal scarring, and no evidence of tissue loss.

At the first postoperative visit, one week after discharge, the dressing was removed (Figures 4A,B**)**. The graft appeared intact, viable, and healing well. Five weeks postoperatively, following completion of 10 HBO sessions, the patient was reevaluated in clinic. The graft had fully integrated with minimal scarring and no evidence of tissue necrosis, retraction, or deformity. The cosmetic result was excellent (Figures 4C,D**)**. The parents reported applying Aquaphor liberally, and the patient remained completely asymptomatic from a urinary standpoint. A follow-up visit was scheduled for three months, and no concerns or complications were reported in the interim.

## 5 Discussion

Although hyperbaric oxygen therapy (HBOT) has primarily been studied in the context of complex hypospadias repairs, its biological mechanisms may be highly relevant in the setting of glans trauma and graft reintegration. Skin grafts rely on adequate oxygen tension and vascular supply from the wound bed, particularly during early stages of healing such as plasmatic imbibition and angiogenesis (3, 4). In cases of compromised vascularity, graft failure is often associated with ischemia, infection, or dehiscence (5, 6). HBOT enhances tissue oxygenation by delivering 100% oxygen under increased atmospheric pressure, promoting capillary angiogenesis, reducing edema, and improving fibroblast and collagen activity (7–11). On a molecular level, HBOT increases vascular endothelial growth factor (VEGF), hypoxia-inducible factor 1-alpha (HIF-1α), and other mediators critical to neovascularization and tissue repair (5–8). Additionally, HBOT suppresses pro-inflammatory cytokines and oxidative stress markers, further stabilizing the graft environment (8–11).

In hypospadias reoperations, HBOT has been shown to improve graft survival, reduce contracture rates, and decrease graft failure (12–14). These mechanisms are directly applicable to glans resurfacing, which similarly depends on vascular support from the corpus spongiosum for graft survival (15). Although pediatric glans resurfacing is rarely reported, traumatic avulsion injuries may warrant similar reconstructive principles.

Children are vulnerable to penile injuries because of the delicate nature and size of their developing genitalia. Common injuries include penile lacerations and scrotal contusions. These injuries arise from various etiologies, including falls, zipper entrapments, and sports-related traumas (1). The management of pediatric penile traumas depends on their severity. Severe penile trauma often requires surgical exploration and staged penile reconstruction with skin grafts and flaps (16).

In our case, the use of adjunctive HBOT following delayed reimplantation of the glans tissue appeared to significantly support full graft integration and avoided tissue necrosis — even with a six-hour delay before revascularization. Prior reports have demonstrated HBOT's role in rescuing post-circumcision glans ischemia in children, further supporting its application in pediatric penile salvage procedures (17, 18).

This case also carries inherent limitations. Most importantly, as a single case report, the findings—while encouraging and clinically successful—cannot be generalized. Patient variability, differences in tissue viability, and unique injury mechanisms mean that an approach effective in one case may not yield similar results in another. Moreover, pediatric penile trauma is a rare entity, and there is a lack of high-level evidence or widely accepted guidelines to inform decisions regarding delayed tissue reimplantation or the use of adjunctive therapies such as hyperbaric oxygen. The decision to proceed with reimplantation and HBOT in this case was guided by clinical judgment and extrapolated experience rather than standardized protocols. Without a control or comparison group, the precise contribution of HBOT to the favorable outcome cannot be definitively determined, though its theoretical and biologic rationale is well supported.

In reviewing the literature, HBOT has primarily been studied in urologic reconstructive contexts such as redo hypospadias surgery, particularly where significant scarring or prior graft failure is present. In those settings, HBOT has been observed to improve graft appearance, reduce fibrosis, and promote healing. For example, several case series have documented improved graft survival and reduced contracture rates in patients receiving HBOT after complex hypospadias repair (12–14). In contrast, literature on traumatic pediatric glans avulsion remains extremely sparse. Most reported cases are managed with primary closure or staged reconstruction, and few describe reimplantation—particularly in a delayed setting (19–21). This case therefore contributes to a limited but growing body of evidence supporting innovative, biologically grounded approaches to complex penile trauma in children, and suggests a potential role for HBOT in selected cases involving partial glans avulsion and tissue salvage.

While our conclusions are based on a single case and informed by extrapolated evidence from a related genitourinary procedure, there are still meaningful strengths to highlight. We were able to closely follow the patient post-operatively, allowing us to observe the graft's integration over time and monitor for any complications. This follow-up provided insight into the potential role of HBOT in promoting graft healing in the setting of pediatric penile trauma.

Nonetheless, these findings suggest HBOT may serve as a valuable adjunct in managing severe pediatric glans injuries, especially in cases of delayed reimplantation where optimal graft oxygenation is critical.

## 6 Conclusion

This case highlights the successful delayed reimplantation of glans tissue in a pediatric patient with partial glans avulsion, supported by adjunctive hyperbaric oxygen therapy. HBOT likely played a critical role in enhancing graft survival by improving tissue oxygenation, promoting angiogenesis, and facilitating wound healing in a context of uncertain graft viability. While further evidence is needed, this case suggests that HBOT may serve as a valuable adjunct in the management of severe pediatric penile trauma involving tissue avulsion and reimplantation.

## Data Availability

The original contributions presented in the study are included in the article/Supplementary Material, further inquiries can be directed to the corresponding author/s.
